# Effect of Supplementing Lysine and Isoleucine as Limiting Amino Acids on Growth Performance and Amino Acid Deposition of *Tenebrio molitor* Larvae Reared on a Cereal-Based Substrate

**DOI:** 10.3390/insects16090989

**Published:** 2025-09-22

**Authors:** Guillermo Fondevila, Habib Fatmi, Pilar Fernando, Carlos Dapoza, Manuel Fondevila

**Affiliations:** 1 Departamento de Producción Animal y Ciencia de los Alimentos, Instituto Agroalimentario de Aragón (IA2), Universidad de Zaragoza-CITA, 50013 Zaragoza, Spain; guillermofondevila@gmail.com (G.F.); fatmihabib48@gmail.com (H.F.); pilarfernando43@gmail.com (P.F.); 2 Evonik Operations GmbH, 08021 Barcelona, Spain; carlos.dapoza@evonik.com

**Keywords:** *Tenebrio molitor* larvae, amino acids, insect feeding, larval growth, protein deposition

## Abstract

The effects of Lys and Ile supplementation in a barley-based substrate on growth performance and nutrient deposition in *Tenebrio molitor* larvae were evaluated. Six trays per treatment containing sixty larvae were assigned to five treatments in two 21-day periods: barley (B); an 85% barley and 15% soybean meal mixture (BS) and B supplemented with synthetic Lys (BL), Ile (BI) or a combination of both (BLI), as potential limiting amino acids (AA) for larval growth. Supplementing AAs was provided in agar cubes twice per week. Larval performance was measured, and larvae were analyzed for nutrient content and AA profile. Final larval weight increased (*p* = 0.042) in BS and BL compared to B, with intermediate values for BI and BLI. Larval contents (mg/larva) in total AAs and seven key AAs (sum of Lys, Met, Cys, Thr, Trp, Val and Ile) were higher in BS-fed larvae than in BL and BLI, with the lowest values for BI and B (*p* < 0.001). Barley supplementation with Lys improved larval AA content, although the levels remained lower than those observed with BS. Conversely, Ile supplementation had no apparent effect on overall AA deposition compared to Lys alone, suggesting the presence of additional limiting AAs for optimal larval growth.

## Simple Summary

Productive performance in the industrial production of *Tenebrio molitor* larvae as a protein source largely depends on an adequate and sufficient supply of essential amino acids (AAs). However, information regarding the AA requirements for the growth of the larvae is limited. The present study evaluated the effects of Lys and Ile supplementation in a barley-based substrate on growth performance and nutrient deposition in *T. molitor* larvae. Larvae were fed barley either alone, combined with soybean meal (85:15), or supplemented with synthetic Lys and Ile in excess, either individually or in combination. Barley supplementation with Lys improved larval growth, as well as protein and AA deposition, although the results remained lower than those observed with substrates based on barley and soybean meal. In contrast, Ile supplementation had no apparent effect on larval performance, and the combination of Lys and Ile did not improve AA production beyond that achieved with Lys alone. These results suggest the importance of Lys as the first limiting AA in barley-based substrates for *T. molitor* larvae, and highlight that other AAs apart from those studied here could also be limiting and impair the efficiency of larval protein production.

## 1. Introduction

Since the onset of the 21st century, the rearing of insects, including *Tenebrio molitor* larvae, has been proposed as a promising protein source for livestock feeds. Several publications have evaluated the amino acid (AA) composition of *T. molitor* larvae [1,2,3]. However, variations in AA proportions have been reported depending on factors such as colony origin, growth stage, environmental factors and feeding conditions [3,4,5]. It is widely accepted that AA profile of *T. molitor* protein is similar to that of soybean meal [6,7], particularly in terms of Lys, Leu, Ala, Met, Thr and Trp contents, but with higher levels of Val and Ile.

The positive influence of dietary protein content on the growth and AA profile of *T. molitor* has been demonstrated [8,9,10]. Quantitatively, Fondevila and Fondevila [11] recommended a minimum level of 12% crude protein (CP, N × 6.25) for substrates based on soybean meal and barley to maximize larval growth, and Mancini et al. [12] recommended a CP level of 11% for substrates based on brewery and bakery by-products for optimal performance. According to theoretical principles, not only the amount of CP but also the AA composition of the protein supplied determines the degree to which larval requirements are satisfied [13,14,15]. In this respect, Plonquet et al. [16] reported that growth performance and protein deposition in *T. molitor* larvae were lower when reared in substrates based exclusively on cereal grains compared to those fed a feed higher in CP and AA, such as wheat bran. This information suggests a potential deficiency in AA availability with respect to larval requirements when fed cereal grains as the sole source of nutrients.

Information regarding the AA requirements for the growth of *T. molitor* larvae is scarce. According to Davis [13], *T. molitor* larvae require the same 10 essential AAs as livestock mammals and poultry, including Lys, Met, Thr, Trp, Val, Ile, Leu, Phe, His and Arg, while Ala, Cys, Pro and Asp could be considered as semi-essential. Using 22 barley cultivars as substrate, Davis and Sosulski [14] suggested that Lys, His, Arg and Thr are positively correlated with larval growth. John et al. [17] emphasized the need for supplementing a dextrin substrate with these limiting AAs in synthetic solid form at the proportions observed in larval tissues to meet growth requirements. Based on the ideal protein concept, the insufficient provision of a specific essential AA in relation to the other dietary AAs would be the limiting factor that determines the maximum potential for protein synthesis, thereby restricting the utilization of the remaining AA, regardless of their concentration. However, antagonisms among AAs such as Ile, Leu and Val, or the influence of the ratios among different essential and non-essential AAs should also be considered to optimize larval growth, as it has been documented for other animal species [18].

Knowledge of the optimal dietary AA profile is crucial to maximize protein accretion and productive performance in *T. molitor* larvae, but information on their specific requirements is currently scarce. The composition of cereal proteins, widely used as substrate ingredients for this insect species, is often deficient in several AA, which can be considered as potentially limiting for growth. Compared with the AA profile of larvae, the protein fraction of barley is likely deficient in Lys and Ile, followed by Thr and Val [2,3,19]. In fact, substrates, including soybean meal or other protein sources, usually result in greater larval performance [16,20,21], but an imbalance in some AAs cannot be absolutely discarded [17]. Identifying and addressing the most limiting AAs in cereal-based substrates could be highly beneficial for improving the efficiency of protein synthesis, ultimately enhancing productivity and sustainability of insect farming systems by optimizing diet formulations. Therefore, the objective of this work was to evaluate the effect of barley supplementation with two synthetic AA, Lys and Ile, on productive performance and AA deposition of *T. molitor* larvae.

## 2. Materials and Methods

The experiment was conducted at the Service for Animal Experimentation of the University of Zaragoza. Animal care and procedures were revised and authorized by the Ethics Committee for Animal Experimentation (PI28/24 NE), who considered that ethical approval was not required; therefore, it was declared as non-evaluable.

### 2.1. Insect Rearing and Experimental Treatments

*T. molitor* larvae at an initial growth stage (approximately 8 weeks after hatching; ranging from 10 to 12 mm length, 37.2 ± 3.71 mg initial weight), previously fed wheat bran, were obtained from our experimental farm and distributed in groups of 60 in plastic trays (15 × 9 × 6 cm). A total of 5 experimental treatments were designed based on the AA availability for growing larvae. Barley grain was used as the basal substrate, offered alone (B) to restrict AA availability; combined with soybean meal at 85:15 ratio to quantitatively and qualitatively improve its AA content (BS) or supplemented with Lys (BL), Ile (BI) or a combination of both (BLI), as potential limiting AA. Two 21-day experimental periods were conducted, with three trays per treatment for each period, resulting in a total of 6 replicates per treatment. Trays were incubated in a climatic chamber (CPR 240 Premium, VWR International Eurolab, Barcelona, Spain), set at 27 °C and 55% relative humidity, and maintained in darkness except during sampling.

Barley and soybean meals were ground to a 2 mm particle size prior to utilization, and substrates were provided at 15 g per tray to ensure substrate availability throughout the 21-day experimental period while avoiding excessive selective behavior. Two agar cubes (3.37 ± 0.23 g, 97% water) were added to each tray twice a week as a source of water. Synthetic AAs (L-Lysine sulfate and L-Isoleucine) were provided at doses sufficient to exceed by 40% the barley supply of Lys and Ile. The chemical composition of substrate ingredients is provided in Table 1. To ensure complete intake and homogenize the supplementation of the test AA throughout the experiment, synthetic Lys and Ile were dissolved in the agar cubes in the corresponding proportions according to treatment and distributed with them. As a result, each cube contained 1.5 mg Lys and/or 1.7 mg Ile. Total CP supply (N × 6.25, mg/initial larvae) per treatment was as follows: B, 26.1 mg; BS, 39.4 mg; BL, 26.2 mg; BI, 26.1 mg and BLI, 26.3 mg. Total Lys and Ile supply (mg/initial larvae) were as follows: B, 0.88 and 0.90 mg; BS, 1.81 and 1.55 mg; BL, 1.18 and 0.90 mg; BI, 0.88 and 1.24 mg and BLI, 1.18 and 1.24 mg.

### 2.2. Measurements

At the end of each experimental period, larvae per tray were counted to calculate larval weight and mortality rate, and both larvae and remaining substrates were weighed to determine larval performance. In particular, larval weight gain, mass gained as the total increase in larval weight per tray; feed intake, as the difference between initial substrate and final residue, and feed-to-gain ratio (F:G), as the amount of substrate consumed per unit of mass gained, were calculated. The estimation of feed intake was not corrected for the frass remaining in the residual substrate, as the finer components of the feed residue could not be separated from the frass by sieving. At the end of each period, larvae per tray were collected, killed by freezing at −80 °C, and lyophilized. Then, larval samples were pooled by treatment and period for further chemical analyses. From these data, nutrient retention in larvae (mg/larva) was estimated by relating composition data and larval weight to calculate dry matter (DM), ether extract (EE), CP and AA deposition.

### 2.3. Laboratory Analyses

Ingredients were analyzed for DM, total ash, CP and EE following the AOAC [22] procedures (methods ref. 934.01, 942.05, ref. 976.05 and ref. 2003.05, respectively). The concentration of neutral detergent fiber was analyzed as described by Mertens [23] in an Ankom 200 Fiber Analyzer (Ankom Technology, Fairport, NY, USA), using α–amylase and sodium sulphite, and results were expressed exclusive of residual ashes. Total starch content was determined enzymatically from samples ground to 0.5 mm using a commercial kit (Total Starch Assay Kit K-TSTA 07/11; Megazyme, Bray, Ireland). The DM content of larvae was determined after lyophilization. Frozen larval samples were ground in a Knifetec 1095 mill (FOSS, Höganäs, Sweden) and analyzed for EE and CP contents by the procedures described above. In addition, larval samples were analyzed for complete AA profile by ion-exchange chromatography (Amino Acid Analyser Biochrom 30+, Cambridge, UK), following the AOAC Official Method ref. 994.12 and regulation (EC) No 152/2009. Briefly, the sample was subjected to oxidation with performic acid-phenol for 16 h at 0 °C, followed by hydrolysis with diluted hydrochloric acid for 24 h at 110 °C. The hydrolysate pH was then adjusted to 2.20 using sodium hydroxide solution before chromatographic separation on a cation exchange resin (sulphonated polystyrene). The separated individual AAs were subsequently reacted with ninhydrin, forming a specific violet dye through post-column derivatization. Photometric detection was performed at a wavelength of 570 nm, or 440 nm for yellow Pro derivatives. In addition, Trp was analyzed separately after alkaline hydrolysis for 20 h at 110 °C.

### 2.4. Statistical Analyses

Data on larval performance and nutrient production among treatments were statistically analyzed as a completely randomized design by ANOVA using the GLM procedure of SAS version 9.4 [24]. Normality of data was verified using the Shapiro–Wilk test prior to analysis, including results on larval mortality. In all cases, the tray was considered as the experimental unit (*n* = 6), and the experimental period was considered as a block. Significant treatment differences were compared using Tukey’s test. Differences with *p* < 0.05 and between *p* = 0.10 and *p* ≥ 0.05 were considered significant or as a trend for significance, respectively.

## 3. Results

### 3.1. Growth Performance

The effects of treatments on larval performance throughout the 21-day experimental period is shown in Table 2. Larval mortality ranged from 7.5 to 13.3% and was not affected by experimental treatments (*p* = 0.306). Initial larval weight did not differ among treatments (*p* = 0.255), but final weight was higher (*p* = 0.042) in BS and BL compared to B, with intermediate values recorded for BI and BLI. As a result, larval growth from 0 to 21 days of experiment was greater (*p* < 0.001) in both BS and BL than in BLI, B and BI. The larval mass produced per tray after 21 days was higher in BS than BLI and B (*p* = 0.012), with BL and BI being intermediate. No differences were detected in substrate intake, and the F:G ratio was better in BS compared to B, BLI and BL, with BI being intermediate (*p* = 0.025).

### 3.2. Nutrient and Amino Acid Deposition

Values on the chemical composition of larvae used to calculate nutrient deposition are shown in Table 3, and the effects of treatment on larval DM, EE and CP retention are shown in Table 4. No differences were observed on DM retention, but EE content in larvae was lower in BS (*p* = 0.019) than in the other treatments. In contrast, CP retention was highest in BS and higher in BL than in B, with BI and BLI being intermediate (*p* < 0.001).

The effects of dietary treatment on larval AA deposition are presented in Table 5. In this respect, AA deposition was greatest (*p* < 0.05) in larvae fed BS than in those fed B, BI, BLI and BL in all cases except for Gly, Ala and Pro, in which no significant differences were detected between BS and BL. In addition, certain variations in statistical significance among treatments were detected for specific AA, with Lys, Cys Ile (*p* < 0.001) and Met + Cys (*p* < 0.01) deposition being greater in larvae fed BL and BLI than in those fed B, with BI showing intermediate results. In fact, the deposition of the sum of total AAs and the seven selected key AAs (sum of Lys, Met, Cys, Thr, Trp, Val, and Ile), was higher for BL than for B, with BLI and BI being intermediate (*p* < 0.001). These results were consistent with those observed for larval contents in Met (*p* < 0.05), as well as in Thr, Arg, Phe, Asp, and Glu (*p* < 0.001). A similar trend was observed for larval deposition of other AAs such as Trp, Val, Leu and Ser, but the contents were significantly lower in larvae fed BI than in those fed BL (*p* < 0.001). Finally, His retention was highest with BS (*p* < 0.01), but differences among the other treatments did not reach significance.

## 4. Discussion

Productive performance in the industrial production of *T. molitor* larvae as a protein source for livestock largely depends on an adequate and sufficient supply of essential AA. In this respect, previous studies highlighted the importance of meeting protein and AA requirements to maximize larval growth Val [10,12,20]. However, some of those studies evaluating the effects of diet composition on growth performance [20,21] and nutrient content [25,26,27] of *T. molitor* larvae used substrates with levels of CP exceeding larval requirements and/or based on multiple and variable combinations of ingredients. In this respect, the wide range of substrates reported in the literature avoids a proper assessment of the specific effect of the AA content in the diet on larval performance, and consequently, larval response to AA availability could not be properly discussed.

In the present study, larvae fed higher CP levels in the BS substrate (15.8%) grew more and showed better F:G ratio than those fed B (10.4%). In general, values reported in the literature suggest that the CP content of the substrate has a positive correlation with growth performance and the CP content of larvae, in agreement with the results reported herein [11,20,21]. For instance, Plonquet et al. [16] observed that the growth performance of *T. molitor* larvae fed 100% wheat bran (17.6% CP in DM basis) was improved compared to larvae fed wheat (10.6% CP), barley (9.1% CP) or corn (7.1% CP) as the sole source of nutrients. However, these differences were significantly reduced when these cereals were combined with different proportions of wheat bran to increase the protein content of the substrate (12.1% CP in DM basis).

The available information regarding the effects of substrate composition on the nutrient and AA content of *T. molitor* larvae is limited. Jankauskiene et al. [28] reported variations in the CP and AA content of larvae fed different combinations of wheat bran and yeast, but no apparent correlations were observed between the AA composition of substrates and larvae. Zhang et al. [29] reported numerical increases in the Leu, Lys, Met + Cys and Thr contents in larvae fed substrates with increasing CP contents (from 3.9 to 17.0%). Other authors [30,31,32] or feed ingredient tables [33,34] reported the complete AA contents in *T. molitor* larvae, but no information regarding the diet fed to the insects was provided. The results observed in the present study indicate that larvae fed B produced less CP and AAs and more EE than those fed BS, probably a result of an increase in the AA and CP supply when soybean meal was included in the diet. Similarly, Bordiean et al. [9] reported that the increase in the CP content of the substrate from 16.6 to 22.8% (DM basis) by the combination of wheat bran with different co-products increased CP and reduced EE deposition in larvae, and Jajić et al. [35] indicated reduced CP and increased EE content in larvae fed barley (11.4% CP in DM basis) compared to those fed wheat bran (20.9% CP) or oats (15.9% CP). Also, Plonquet et al. [16] observed that the use of barley-based substrates (9.1% CP in DM basis) increased the EE content at the expense of CP in *T. molitor* larvae compared to those fed diets based on wheat bran (17.6% CP) or wheat grain (10.6% CP). In the same research [16], it was reported that the CP content of larvae increased from 38.4 to 44.5% (DM basis), and the EE content decreased from 44.1 to 33.8%, when barley grain substrate was combined with wheat bran to reach 12.1% CP. The reasons for the increase in the lipid fraction content in larvae fed barley could be associated with a relatively higher starch and lower CP contents compared to other ingredients but also could be related to a suboptimal AA supply, which would potentially limit protein production, favoring fat deposition. In any case, these results indicate that potential impairments in larval development fed substrates with limited AA content, when using barley as the sole substrate, could be overcome by the supplementation with AA-rich ingredients such as soybean meal.

The effects of substrate supplementation with synthetic AAs on the growth performance of *T. molitor* larvae have previously been studied using semi-synthetic diets, with AAs provided in solid form [15]. In the context of that study, a potential rejection by the larvae of synthetic AAs because of their crystalline structure might reduce their effective intake, which in turn would limit AA availability and impair growth performance. In the present study, the agar cubes, offered both as a water source for the larvae and as a vehicle for the supplementation of synthetic AAs, were fully consumed between distributions throughout the trial period. This information ensures that the synthetic AA supplemented, dissolved in the water source, were completely and continuously consumed, and suggests that this methodology is a proper strategy for evaluating the effects of nutrient supplementation in *T. molitor* larvae in future research, provided that the compounds tested are soluble in water.

Early studies [36,37] aiming to calculate AA requirements in *T. molitor* larvae indicated that substrate supplementation with a mixture of AAs based on larval protein composition improved growth performance in larvae fed a glucose/dextrose basal diet. Based on nutritional principles, a low supply of protein and AAs in the diet contributes to the potential deficiency of a particular limiting AA, thus decreasing the utilization of other AAs and reducing larval growth and protein deposition [18]. Once the requirements for the first limiting AA are satisfied, protein deposition is expected to increase until the availability of a second limiting AA restricts further protein retention. In the present study, the supplementation of B with synthetic Lys resulted in similar larval growth to that observed in larvae fed BS. Furthermore, supplementation of B with synthetic Lys increased CP deposition as well as the retention of most AAs (e.g., Lys, Met, Cys, Thr, Trp, Val and Ile) in larvae, with a numerical reduction in the EE content, with values that were eventually similar (e.g., Gly, Ala and Pro) to those obtained with the BS diet. Similarly, the role of Lys in promoting growth and protein accretion in larvae reared on barley substrates has been previously reported [14]. This information suggests that, based on the ideal protein concept that is widely accepted in other productive species such as poultry and pigs, Lys could be considered as the first limiting AA for the growth of *T. molitor* larvae reared on barley-based substrates. Therefore, the additional supply of Lys, as the first limiting AA in cereal-based diets, would allow a more balanced AA profile of the substrate, favoring productive performance and protein deposition. In contrast, supplementation of B with Ile did not significantly affect larval weight gain or protein and AA production, and in fact, the combined supplementation of Lys and Ile did not improve the results obtained compared to Lys alone. In this respect, although treatment BLI numerically increased larval CP and AA deposition compared to treatment B, these differences were not statistically significant in some cases. However, overall nutrient deposition in larvae fed BLI was similar to that observed in larvae fed BL. Consequently, the lack of statistical significance between BLI and B treatments may be attributed to a limitation in statistical power rather than the absence of a biological effect. The lack of effect observed with Ile supplementation (provided either alone or in combination with Lys) might be attributed to a limited ability of the larvae to use the synthetic Ile. However, this explanation is doubtful considering the high digestibility of synthetic Ile in swine and poultry. Also, interactions among branched-chain AAs (Leu, Ile and Val) are known to modify AA requirements and growth performance in different animal species, as a result of the competition for similar metabolic pathways [38,39]. However, a limited Ile availability resulting from increased Leu or Val supply in the current research was discarded due to the low contents in these AAs in barley grain. In this regard, Davis and Sosulski [14] did not observe any significant correlation between the Ile content of the substrate and growth performance and protein retention of *T. molitor* larvae fed 22 different batches of barley. The information provided suggests that, despite the significant differences in Ile content between the protein fraction of barley and that of the larvae, the presence of other limiting AAs not considered in this research may limit larval growth and protein production in barley-based substrates.

## 5. Conclusions

The results reported in the current research indicate that the use of substrates based on barley as the sole source of nutrients do not provide the amount of AAs required for optimal growth of *T. molitor* larvae. In fact, increasing the dietary CP content from 10.4 to 15.8% by the combination of barley with soybean meal increased protein and AA retention and decreased EE deposition per larva. Probably, the presence of certain limiting AAs in cereal grains such as barley might contribute to the impairments in larval performance and protein deposition observed in the current study. On the other hand, barley supplementation with synthetic Lys improved larval performance and AA content, although the levels generally remained lower than those observed in larvae fed BS. In contrast, Ile supplementation had no apparent effect on overall AA deposition. These results suggest that Lys is the first limiting AA in barley-based substrates for the production of *T. molitor* larvae, and the lack of effect observed by Ile supplementation indicates the presence of additional limiting AAs for optimal growth. The information provided confirms the importance of a balanced and sufficient supply of AAs to optimize larval development and could be valuable to be used in practice to formulate complete diets for *T. molitor* larvae. In this respect, the assessment of the most limiting AAs in cereal-based substrates is essential to prepare adequate combinations of ingredients to supply sufficient AAs to optimize larval performance. Future research should be oriented to identify other limiting AAs in cereal-based substrates and to provide accurate information on the minimum requirements to optimize larval composition and performance.

## Figures and Tables

**Table 1:** Chemical composition (g/kg) of the tested ingredients.

	Barley	Soybean Meal
Dry matter	890	887
Ash	134	184
Crude protein	104	461
Ether extract	10	18
Neutral detergent fiber	262	94
Starch	533	11
Amino acids		
Alanine	4.3	20.3
Arginine	5.3	33.4
Aspartic acid	6.5	51.9
Cystine	2.4	6.4
Glutamic acid	23.6	81.0
Glycine	4.4	19.6
Histidine	2.3	12.0
Isoleucine	3.6	21.0
Leucine	7.2	34.9
Lysine	3.5	28.5
Methionine	1.8	6.0
Phenylalanine	5.2	23.4
Proline	11.1	22.7
Serine	4.4	23.4
Threonine	3.6	18.3
Tryptophan	1.4	6.0
Valine	5.2	22.1
7 key amino acids ^1^	21.5	108.3
Total amino acids	95.8	430.9

^1^ Sum of Lysine, Methionine, Cystine, Threonine, Tryptophan, Isoleucine and Valine.

**Table 2:** Mortality, growth performance, feed intake and feed-to-gain ratio (F:G) in *T. molitor* larvae fed barley alone (B), an 85:15 barley:soybean meal mixture (BS) or barley supplemented with Lys (BL), Ile (BI) or a combination of both amino acids (BLI) for 21 days (*n* = 6).

	B	BS	BL	BI	BLI	SEM	*p*-Value
Mortality (%)	13.33	9.72	11.94	7.50	10.28	1.963	0.306
Initial weight (mg/larva)	35.3	35.6	38.3	38.8	38.3	1.40	0.255
Final weight (mg/larva)	116 ^b^	125 ^a^	125 ^a^	119 ^ab^	120 ^ab^	2.4	0.042
Larval growth (mg/d)	3.82 ^b^	4.26 ^a^	4.11 ^a^	3.81 ^b^	3.87 ^b^	0.077	<0.001
Larval mass (mg/tray)	3882 ^b^	4623 ^a^	4253 ^ab^	4237 ^ab^	4128 ^b^	132.9	0.012
Feed intake (mg/tray)	6098	6340	6378	6212	6238	103.4	0.358
F:G (mg/mg)	1.578 ^a^	1.379 ^b^	1.505 ^a^	1.467 ^ab^	1.514 ^a^	0.0396	0.025

SEM: standard error of means. Within rows, when *p* < 0.05 values not sharing the same letter are significantly different.

**Table 3:** Analyzed ^1^ chemical and amino acid composition (mg/g dry matter unless indicated) of *T. molitor* larvae fed barley alone (B), an 85:15 barley:soybean meal mixture (BS) or barley supplemented with Lys (BL), Ile (BI) or a combination of both amino acids (BLI) for 21 days.

	B	BS	BL	BI	BLI
Dry matter (mg/g fresh matter)	363.5	357.4	360.7	359.4	362.6
Ether extract	425.0	367.4	416.4	418.9	413.1
Crude Protein ^2^	424.1	477.2	430.3	425.5	435.4
Amino acids (AAs)					
Alanine	28.34	29.74	28.67	28.07	28.48
Arginine	20.26	23.42	20.73	20.68	21.05
Aspartic acid	28.96	34.68	30.13	29.40	30.02
Cystine	3.80	4.41	3.94	3.87	4.02
Glutamic acid	42.04	50.72	43.65	42.83	43.97
Glycine	19.89	22.08	20.51	20.00	20.36
Histidine	11.29	12.91	11.40	11.43	11.43
Isoleucine	16.22	18.84	16.66	16.61	17.11
Leucine	26.87	31.20	27.67	26.95	27.81
Lysine	20.06	24.21	21.34	20.70	21.54
Methionine	4.77	5.51	4.93	4.91	4.97
Methionine + Cystine	8.57	9.92	8.87	8.79	8.99
Phenylalanine	12.14	14.71	12.40	12.30	12.54
Proline	30.80	32.57	32.42	32.91	32.87
Serine	16.91	19.01	17.41	16.90	17.24
Threonine	14.40	16.60	14.88	14.54	14.81
Tryptophan	4.81	5.86	4.91	4.73	4.94
Valine	23.10	25.64	23.57	22.92	23.36
Σ 7 key AA ^3^	87.2	101.1	90.2	88.3	90.8
Total AA	325	372	335	330	337

^1^ *n* = 6 for dry matter and *n* = 2 for ether extract, crude protein and amino acid analysis ^2^ N × 6.25 ^3^ Sum of Lysine, Methionine, Cysteine, Threonine, Tryptophan, Isoleucine and Valine.

**Table 4:** Dy matter, ether extract and crude protein (N × 6.25) retention (mg/larva) in *T. molitor* larvae fed barley alone (B), an 85:15 barley:soybean meal mixture (BS) or barley supplemented with Lys (BL), Ile (BI) or a combination of both amino acids (BLI) for 21 days (*n* = 6).

	B	BS	BL	BI	BLI	SEM	*p*-Value
Dry matter	42.01	44.65	44.93	42.70	43.40	1.045	0.255
Ether extract	17.85 ^a^	16.40 ^b^	18.71 ^a^	17.90 ^a^	17.93 ^a^	0.439	0.019
Crude protein	17.82 ^c^	21.31 ^a^	19.33 ^b^	18.17 ^bc^	18.90 ^bc^	0.468	<0.001

SEM: standard error of means. Within rows, when *p* < 0.05 values not sharing the same letter are significantly different.

**Table 5:** Amino acid (AA) retention (mg/larva) in *T. molitor* larvae fed barley alone (B), an 85:15 barley:soybean meal mixture (BS) or barley supplemented with Lys (BL), Ile (BI) or a combination of both amino acids (BLI) for 21 days (*n* = 6).

	B	BS	BL	BI	BLI	SEM	*p*-Value
Alanine	1.190 ^c^	1.328 ^a^	1.288 ^ab^	1.199 ^bc^	1.236 ^ab^	0.0321	0.025
Arginine	0.851 ^c^	1.045 ^a^	0.931 ^b^	0.885 ^bc^	0.914 ^bc^	0.0250	<0.001
Aspartic acid	1.216 ^c^	1.548 ^a^	1.353 ^b^	1.258 ^bc^	1.303 ^bc^	0.0042	<0.001
Cysteine	0.160 ^c^	0.197 ^a^	0.177 ^b^	0.166 ^bc^	0.175 ^b^	0.0042	<0.001
Glutamic acid	1.795 ^c^	2.264 ^a^	1.961 ^b^	1.832 ^bc^	1.909 ^bc^	0.0535	<0.001
Glycine	0.835 ^c^	0.986 ^a^	0.922 ^ab^	0.855 ^bc^	0.884 ^bc^	0.0231	<0.001
Histidine	0.474 ^b^	0.576 ^a^	0.512 ^b^	0.489 ^b^	0.496 ^b^	0.0135	<0.001
Isoleucine	0.681 ^c^	0.841 ^a^	0.748 ^b^	0.710 ^bc^	0.743 ^b^	0.0188	<0.001
Leucine	1.129 ^c^	1.939 ^a^	1.243 ^b^	1.151 ^c^	1.207 ^bc^	0.0313	<0.001
Lysine	0.842 ^c^	1.080 ^a^	0.958 ^b^	0.887 ^bc^	0.935 ^b^	0.0250	<0.001
Methionine	0.200 ^c^	0.246 ^a^	0.221 ^b^	0.210 ^bc^	0.216 ^bc^	0.0059	0.017
Methionine + Cystine	0.360 ^c^	0.443 ^a^	0.398 ^b^	0.375 ^bc^	0.390 ^b^	0.0100	<0.01
Phenylalanine	0.510 ^c^	0.657 ^a^	0.557 ^b^	0.526 ^bc^	0.544 ^bc^	0.0148	<0.001
Proline	1.291	1.453	1.456	1.413	1.428	0.0412	0.053
Serine	0.710 ^c^	0.849 ^a^	0.782 ^b^	0.722 ^c^	0.748 ^bc^	0.0176	<0.001
Threonine	0.605 ^c^	0.741 ^a^	0.668 ^b^	0.621 ^bc^	0.643 ^bc^	0.0168	<0.001
Tryptophan	0.202 ^c^	0.262 ^a^	0.221 ^b^	0.202 ^c^	0.215 ^bc^	0.0060	<0.001
Valine	0.970 ^c^	1.144 ^a^	1.059 ^b^	0.980 ^c^	1.123 ^bc^	0.0260	<0.001
Σ 7 key AA ^1^	3.660 ^c^	4.511 ^a^	4.053 ^b^	3.776 ^bc^	3.940 ^bc^	0.1025	<0.001
Total AA	13.43 ^c^	16.35 ^a^	14.84 ^b^	13.90 ^bc^	14.40 ^bc^	0.384	<0.001

^1^ Sum of Lysine, Methionine, Cysteine, Threonine, Tryptophan, Isoleucine and Valine. SEM: standard error of means. Within rows, when *p* < 0.05 values not sharing the same letter are significantly different.

## Data Availability

Data are contained within the article.

## Abbreviations

The following abbreviations are used in this manuscript:

CP	Crude protein (N × 6.25)
EE	Ether extract
DM	Dry matter
F:G	Feed to Gain ratio
SEM	Standard error of means
